# Prevalence of suicidal ideation, suicidal attempt and completed suicide in Ethiopia: a systematic review and meta-analysis protocol

**DOI:** 10.1186/s13643-019-0986-8

**Published:** 2019-03-22

**Authors:** Berhanu Boru Bifftu, Berihun Assefa Dachew, Bewket Tadesse Tiruneh, Yonas Deressa Guracho

**Affiliations:** 1 University of Gondar College of Medicine and Health Science, School of Nursing, Gondar, Ethiopia; 2 Department of Epidemiology and Biostatistics, Institute of Public Health, College of Medicine and Health Sciences, University of Gondar, Gondar, Ethiopia; 3 The University of Queensland, Institute for Social Science Research, Indooroopilly, QLD 4068 Australia; 4 Department of Psychiatry, Bahir Dar Health Science College, Bahir Dar, Ethiopia

**Keywords:** Ethiopia, Completed suicide, Suicidal behaviors, Suicidal ideation, Suicidal attempt

## Abstract

**Background:**

Suicide is an emergency psychiatric problem that needs an urgent intervention. Ethiopia, as a nation, has been experiencing high burden of suicide. Cultural taboo, stigma, and criticism associated with suicidal behaviors affect the communities’ attitude and practice toward suicide and its help-seeking behaviors. Thus, this systematic review and meta-analysis will provide the pooled prevalence of suicidal ideation, suicidal attempt, and completed suicide in Ethiopia.

**Methods:**

This review protocol is designed according to the Preferred Reporting Items for Systematic Reviews and Meta-Analysis Protocols (PRISMA-P 2015 Guidelines). Studies reporting the prevalence of suicidal ideation, suicidal attempt, and completed suicide will be included from the databases of PubMed/MEDLINE, Cochrane Library, SCOPUS, EMBASE, and Web of Sciences. The reference lists of retrieved articles, Google Scholar, and national health database reporting suicide will be also searched. No time and language restrictions will be imposed on the search strategy. The methodological quality of included studies will be assessed using the Joanna Briggs Institute critical appraisal tool. The heterogeneity between studies will be measured by the index of heterogeneity (*I*^2^ statistics) test. Funnel plots and Egger’s test will be used to determine publication bias. Moreover, subgroup and sensitivity analyses will be carried out. The pooled prevalence of suicidal ideation, suicidal attempt, and completed suicide will be calculated by metaprop command using random effects model with the Dersimonian and Laird method.

**Discussion:**

This systematic review and meta-analysis aims to explore the available epidemiological evidences about suicidal ideation, suicidal attempt, and completed suicide. The final results of this review will be submitted for publication in a peer-reviewed journal and presented at conferences. The review of the results will help to raise awareness about the burden of suicidal ideation, suicidal attempt, and completed suicide in Ethiopia.

**Systematic review registration:**

PROSPERO CRD42018112836

## Background

Suicide is a complex process ranging from suicidal ideation to planning of suicide, suicidal attempt, and completed suicide [1, 2]. Two overlapping terms are used to describe the concept of suicide [1, 3, 4]. These are (i) suicidal attempt/self-harm/suicidal intent, refers to a deliberated direct destruction of body tissues with a conscious suicidal intent, and (ii) non-suicidal attempt/self-injury (NSSI), refers to a deliberated direct destruction of body tissues without a conscious suicidal intent [1, 3, 4]. Most standard guidelines focus on self-harm irrespective of the intent, and there are commonly four different forms of suicidal behaviors [1, 3]. These are (i) suicidal ideation, refers to thoughts fantasies and wishes about ending of one’s own life, (ii) suicide plan, refers to planning on how to end one’s own life, (iii) suicide attempt, refers to the self-destructive act with intent to end one’s own life, and (iv) completed suicide, refers to the act of self-harm with a fatal outcome [1–3]. Suicide is a worldwide phenomenon that takes a heavy toll on the individuals, families, and communities [5]. Globally, one million people die due to suicide per year (one person every 40 s) [5–9]. It is the tenth leading cause of death worldwide, but the second and third leading cause for those age group between 15 and 29 years and 15–44 years respectively [1, 7, 9–12]. It is predicted that, by 2020, annual suicide fatalities will be increased to 1.5 million (one every 20 s) [5]. According to the World Health Organization (WHO) study among 21 countries, approximately, the 12 months and lifetime prevalence of suicidal attempt was 0.3 to 0.4% respectively 2 and 9% for suicidal ideation [10]. Suicidal attempt is more common than suicide deaths [5, 8]. For example, in the USA, there are more than 30 suicidal attempts for each suicide death [10]. In Africa, the overall magnitude of suicide ranged from 2–3% [range 0.7–6.0%] [11, 13]. In Ethiopia, the prevalence of suicidal behaviors ranged from 0.9 to 60% [14–16] for suicidal ideation, 3.8 to 27% [14, 17] for suicidal attempt, and 13.96% per 100,000 [18, 19] for completed suicide.

Suicidal ideation, plan, and attempt are major risk factors for completed suicide [1, 5]. Among individual with lifetime suicidal ideation, the probability of ever making suicidal plan was 33%, and attempted suicide was 30%. For lifetime history of suicidal ideation and plan, the probability of completed suicide was 55% [8]. Factors such as psychiatric disorder [20], hopelessness, and substance use were also associated with completed suicide [1, 3, 5, 20]. Suicide is an emergency psychiatric problem [9, 20, 21] and need an urgent treatment; yet, stigma, taboo, laws, and other associated cultural criticism affect the societies’ attitude and practice toward the identification and treatment of suicidal behaviors [3, 5, 8, 9, 13, 21]. In spite of the high burden of suicidal behaviors, developing countries [11, 13] like Ethiopia give little research attention, and the available individual studies also reported inconsistent results, which need comprehensive up-to-date information. Thus, the aim of this proposed systematic review and meta-analysis is to assess the prevalence of suicidal ideation, suicidal attempt, and completed suicide in Ethiopia.

## Methods

This protocol is designed according to the Preferred Reporting Items for Systematic Reviews and Meta-Analysis Protocols (PRISMA-P 2015 Guidelines) [22]. This review protocol has been registered in the PROSPERO, International Prospective Register of Systematic Reviews, with the registration number of CRD42018112836.

### Search strategy

The search and document retrieval strategy will be intended to capture range of published and unpublished literature using databases including PubMed/MEDLINE, Cochrane Library, SCOPUS, EMBASE, and Web of Sciences. A combination of Medical Subject Headings (MeSH) thesaurus, text words, and combining with appropriate Boolean operators will be used in order to identify as many studies as possible. We will develop a comprehensive search of MEDLINE through PubMed search strategy tailored to each database. No time and language restrictions will be imposed on the search strategy. Reference lists of all articles will also be searched. Moreover, national health databases reporting suicide including the Ethiopian Journal of Health Development (EJHD) (1984) (http://www.ejhd.org/index.php/ejhd/index), Ethiopian Journal of Health Science (EJHS) (1990) (https://www.ju.edu.et/ejhs/), and the Central Statistical Agency (CSA) (www.csa.gov.et) will be searched. Furthermore, Google Scholar will be searched for gray literature. The drafted full electronic search strategy for PubMed/MEDLINE database is included in the supplementary information.

### Selection of studies

All articles retrieved through search strategy will be imported to EndNote X7 (Thomson Reuters, New York, USA). After excluding the duplicated studies from EndNote Library, the title and abstracts of the remaining articles will be assessed independently by two reviewers (BBB and YDG) and disagreement will be resolved by discussion and/or third author (BTT). Full-text studies will be included in the systematic review and meta-analysis. Conference abstracts, letters to editors, review, and commentary articles will be excluded.

### Definition of concepts

(i) Suicide is defined as an act of self-harm with a fatal outcome, (ii) deliberate self-harm is a non-fatal act of self-harm carried out with variable motivations, (iii) suicidal ideation is the engagement in thoughts about self-inflicted harm to end one’s own life, (iv) suicidal plan is the formulation of a specific method through which one intends to die, (v) suicidal attempt is the engagement in potentially self-injurious behaviors in which there is at least some intent to die, and (vi) suicidal behaviors include both fatal and non-fatal suicidal behaviors [1–3].

### Eligibility criteria

#### Participants

This review will targets all human participants that reported the prevalence of suicidal ideation, suicidal attempt, and completed suicide irrespective of age, sex, setting (institution [health facility/school (high school, college, and university)] and community), and population (patient, general population). Studies in which participants are drawn from prisons, refugees, and homeless will be excluded because they are not the representative of either patient or the general population.

#### Outcome measures

This review will include studies that investigated the prevalence of suicidal ideation, suicidal attempt, and completed suicide using diagnostic criteria in the Tenth Revision of the International Statistical Classification of Diseases and Related Health Problems (ICD-10) or the Diagnostic and Statistical Manual of Mental Disorders (DSM)-IV or DSM-5) or autopsy or chart review or self-developed tool. All available studies, irrespective of the data collection tools or cutoff scores and duration (current, period (6 months or 12 months), or lifetime prevalence), will be included in the systematic review and meta-analysis.

### Study design

Observational studies (cross-sectional and cohort/longitudinal) will be included in this systematic review and meta-analysis. Studies that focused on case reports and conference abstracts with inadequate information will be excluded.

### Data extraction

Data will be extracted from the eligible studies by two independent reviewers (BBB and YDG) using a pre-conceived data abstraction form, and any disagreement will be resolved by discussion and/or by the third author (BTT). The extracted data will be collated in a Microsoft Excel 2016 spreadsheet (Microsoft, Redmond, Washington, USA). These data will include name of first author last name, year of publication, study setting/region, study design, study population, age and sex, sample size, response rate, data collection tool, prevalence of suicidal ideation, suicidal attempt, and completed suicide.

### Quality assessment

The methodological quality of included studies will be assessed by two independent reviewers (BBB and YDG) using the Joanna Briggs Institute Meta-analysis of Statistics Assessment and Review Instrument (JBI-MAStARI) critical appraisal tool for prevalence studies [23]. Disagreements will be solved by discussion and/or by the third author (BTT). The JBI quality assessment tool for prevalence studies has eight items with response option “yes,” “no,” “unclear,” and “not applicable”. The results of each individual paper will be graded with a score ranging from 0 to 8. Interpersonal scoring discrepancies during critical appraisal will be resolved after a thorough discussion, and if disagreement will be continued, the decision of the quality assessment score will be based on the calculated mean scores of the three reviewers. All articles scored greater than 50% will be included in the analysis.

### Data synthesis and statistical analysis

The extracted data from the eligible studies will be entered into a Microsoft Excel Database and then will be imported into STATA version 14 (Stata Corp LLC, Texas, USA) for analysis. The pooled prevalence of suicidal ideation, suicidal attempt, and completed suicide will be calculated by metaprop command using a random effects model [24] with the Dersimonian and Laird method based on the transformed values and their variance [25]. We will fit the Freeman–Tuckey variant of the arcsine square root transformation of proportions to avoid variance instability when handling proportions close to one [26]. The magnitude of heterogeneity between studies will be measured by the index of heterogeneity (*I*^2^ statistics) test [27]. *I*^2^ values of 25%, 50%, and 75% will be used as low, medium, and high heterogeneity respectively. Generally, we plan to conduct subgroup analyses by sex, age, sample size, study quality (high, fair, and low), setting (institution and community based), year of publication, time frame (lifelong and last 12 months), and study population (patient, general community, and students). Publication bias will be evaluated using the visual funnel plot [28] and Egger test [29]. A *p* value < 0.1 will be considered as indicative of statistically significant publication bias. If there is evidence of small study effect and heterogeneity, we plan to perform sensitivity analysis [30].

### Presenting and reporting results

A flow diagram will be included to outline the step by step process of study selection methods. The characteristics and quality assessment of the included studies will be presented in tables. Pooled estimates will be presented using forest plots.

## Discussion

To the best of the authors’ knowledge, there is no systematic review and meta-analysis has been reported about the suicidal ideation, suicidal attempt, and completed suicide in Ethiopia. This needs a comprehensive epidemiological evidence for further research and decision-making for the prevention and control of suicide. Thus, this systematic review and meta-analysis will explore the available epidemiological evidences about suicidal ideation, suicidal attempt, and completed suicide in Ethiopia. The results of this systematic review and meta-analysis will serve to draw attention and raise awareness among policy maker, health care practitioners, and researchers on the growing epidemiological concern of suicidal ideation, attempted suicide, and completed suicide in Ethiopia. This study will be based on published data; thus, approval of ethical clearance will not be required. The final report of the systematic review and meta-analysis will be disseminated in the form of a scientific paper (published) in a peer-reviewed journal. Moreover, the results will be presented at conferences and submitted to relevant health authorities. We plan to update the review in the future to evaluate the changes in epidemiological evidences.

### Strength and potential limitations

This review of protocol is the first systematic review and meta-analysis protocol about suicidal ideation, suicidal attempt, and completed suicide in Ethiopia. The registration of review protocol in the PROSPERO, International Prospective Register of Systematic Reviews, adheres to the Preferred Reporting Items for Systematic Reviews and Meta-Analyses protocol (PRISMA-P 2015 guideline). Plans to include studies without the restriction of the publication year and to perform tests for heterogeneity, sensitivity analysis, and subgroup analysis will help to get the desired results. The exclusion of studies carried out among prisoners and refugees will minimize the number of included studies, and there will be a possibility of missing unpublished studies.

## Abbreviations

CI: Confidence interval
CRD: Centre for Reviews and Dissemination
DSH: Deliberate self-harm
DSM-IV: Diagnostic and Statistical Manual of Mental Disorders 4th edition
EMBASE: Excerpta Medica database
*I*^2^: Index of heterogeneity
ICD-10: Tenth Revision of the International Statistical Classification of Diseases and Related Health Problems
MEDLINE: Medical Literature Analysis and Retrieval System Online
MeSH: Medical Subject Headings
NSSI: Non-suicidal self-injury
OR: Odds ratio
PRISMA-P: Preferred Reporting Items for Systematic Review and Meta-Analysis Protocols
